# Combination of OipA, BabA, and SabA as candidate biomarkers for predicting *Helicobacter pylori*-related gastric cancer

**DOI:** 10.1038/srep36442

**Published:** 2016-11-07

**Authors:** Yu-Lin Su, Hsiang-Ling Huang, Bo-Shih Huang, Po-Chung Chen, Chien-Sheng Chen, Hong-Long Wang, Pin-Hsin Lin, Meng-Shu Chieh, Jiunn-Jong Wu, Jyh-Chin Yang, Lu-Ping Chow

**Affiliations:** 1 Graduate Institute of Biochemistry and Molecular Biology, College of Medicine, National Taiwan University, Taipei, Taiwan; 2 Graduate Institute of Systems Biology and Bioinformatics, National Central University, Taoyuan, Taiwan; 3 Department of Statistics, National Taipei University, New Taipei City, Taiwan; 4 First Core Laboratory, College of Medicine, National Taiwan University, Taipei, Taiwan; 5 Department of Medical Laboratory Science and Biotechnology, Center of Infectious Disease and Signaling Research, College of Medicine, National Cheng Kung University, Tainan, Taiwan; 6 Department of Internal Medicine, Hospital and College of Medicine, National Taiwan University, Taipei, Taiwan; 7 Center of Genomic Medicine, National Taiwan University, Taipei, Taiwan

## Abstract

*Helicobacter pylori (H. pylori* ) infection is a major cause of chronic gastritis and is highly related to duodenal ulcer (DU) and gastric cancer (GC). To identify *H. pylori*-related GC biomarkers with high seropositivity in GC patients, differences in levels of protein expression between *H. pylori* from GC and DU patients were analyzed by isobaric tag for relative and absolute quantitation (iTRAQ). In total, 99 proteins showed increased expression (>1.5-fold) in GC patients compared to DU patients, and 40 of these proteins were categorized by KEGG pathway. The four human disease-related adhesin identified, AlpA, OipA, BabA, and SabA, were potential GC-related antigens, with a higher seropositivity in GC patients (n = 76) than in non-GC patients (n = 100). Discrimination between GC and non-GC patients was improved using multiple antigens, with an odds ratio of 9.16 (95% CI, 2.99–28.07; p < 0.0001) for three antigens recognized. The optimized combination of OipA, BabA, and SabA gave a 77.3% correct prediction rate. A GC-related protein microarray was further developed using these antigens. The combination of OipA, BabA, and SabA showed significant improvement in the diagnostic accuracy and the protein microarray containing above antigens should provide a rapid and convenient diagnosis of *H. pylori*-associated GC.

*Helicobacter pylori (H. pylori*), a Gram-negative spiral-shaped bacterium that selectively colonizes the human gastric mucosa, infects approximately half of the world’s population. Although the majority of infected patients remain asymptomatic, *H. pylori* infection is associated with the development of a variety of gastric diseases, such as chronic active gastritis (GS), peptic ulcer, gastric MALT lymphoma, and gastric cancer (GC)^1^,^2^. *H. pylori* is also recognized as a class I carcinogen and is considered the most common etiologic agent of infection-related cancers^3^.

*H. pylori* infection is thought to be involved in the development of both GC and duodenal ulcer (DU), which are at opposite ends of the disease spectrum, since it is well documented that patients with DU rarely develop GC^4^,^5^. The involvement of *H. pylori* infection in both of these gastroduodenal diseases might be explained by considering various host factors, duration of infection, environmental factors, dietary factors, and the presence of different bacterial virulence factors^6^,^7^. *H. pylori* is genetically highly variable, and this variability includes the production of different *H. pylori* virulence factors with different degrees of pathogenicity^3^,^8^. A number of virulence factors of *H. pylori* have been proposed for use in predicting the development of different gastroduodenal diseases. Of these, vacuolating toxin A (VacA) and cytotoxin-associated gene A (CagA) have been suggested as possible risk factors for peptic ulcer and GC^9^,^10^,^11^. Other studies have demonstrated that duodenal ulcer-promoting gene A (DupA) of *H. pylori* is a DU-related virulence factor^12^,^13^.

GC remains the fourth most common cancer and is the second leading cause of cancer-related deaths worldwide^14^. However, there are still no reliable diagnostics to predict the risk for developing GC among *H. pylori*-infected patients^15^,^16^. Several virulence factors of *H. pylori* may contribute to gastric carcinoma pathogenesis^8^,^17^,^18^. A strong humoral immune response to *H. pylori* antigens is elicited by *H. pylori*-induced infiltration of the mucosa by neutrophils, lymphocytes, and macrophages^19^,^20^ and *H. pylori-*induced antibody has been used in clinical serologic screening^14^,^15^,^21^. GC-related virulence factors of *H. pylori* may are therefore potential candidates for biomarkers, and would be of benefit in serological screening for detecting, and predicting the outcome of, *H. pylori* infection. Several *H. pylori* virulence antigens have been suggested for use in serologic screening for GC^10^,^22^,^23^,^24^. However, the ability to discriminate between GC and non-GC diseases is still poor and a better method is required to improve the efficacy of GC detection.

Proteomic technologies based on mass spectrometry have been increasingly used in the search for diagnostic and prognostic biomarkers in GC^25^,^26^,^27^,^28^. Protein microarrays have also been demonstrated to be an effective high throughput platform for the clinical diagnosis of various diseases^29^. In this study, we used a quantitative isobaric tag for relative and absolute quantitation (iTRAQ) proteomic approach to identify GC-related virulence factors of *H. pylori* by comparing *H. pylori* proteins that are differentially expressed in GC and DU patients. Four adhesins, adherence-associated lipoprotein A (AlpA), outer inflammatory protein A (OipA), blood group antigen-binding adhesin A (BabA), and sialic acid-binding adhesin A (SabA), were identified and were more frequently recognized by GC sera than by DU sera. Statistical analysis of the serology data showed that the ability of discrimination was greater when three of the four antigens OipA, BabA, and SabA were used in combination. We also developed a GC-related protein microarray that can simultaneously detect serum antibody to these three GC-related antigens which can be used to distinguish between *H. pylori*-infected GC patients with different gastric diseases.

## Results

### Identification and quantification of differentially-expressed proteins in H. pylori strains from patients with GC or DU by iTRAQ analysis

**Figure 1:**
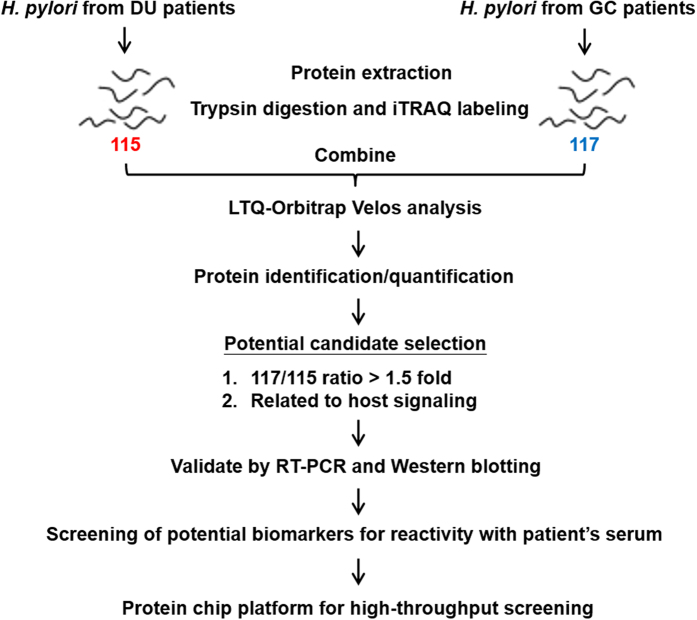
Flow diagram for the identification of differentially expressed virulence factors in different *H. pylori* strains. Proteins from pooled *H. pylori* strains from DU patients or GC patients (n = 5 per group) were digested and labeled separately with different iTRAQ reagents in three independent repeats, then the labeled peptides were combined and separated on a LTQ-Orbitrap Velos hybrid mass spectrometer. Increased expression (>1.5-fold) in GC-derived *H. pylori* strains than in DU-derived *H. pylori* strains and proteins related to host signaling by KEGG pathway mapping were used as criteria for selection of candidates, and the candidates were validated by RT-PCR and Western blotting. The selected candidates were then verified by reaction with patients’ serum samples, and a GC-related protein microarray platform was established to perform rapid diagnosis testing.

GC and DU are at the opposite ends of the disease spectrum and *H. pylori* strains from GC or DU patients expressing different bacterial virulence proteins might be one of the factors determining the outcome of *H. pylori* infection^4^. To detect differentially expressed proteins in *H. pylori* in GC patients and DU patients, quantitative iTRAQ labeling-based proteomic analysis was performed. A schematic diagram of the experimental design is shown in Fig. 1. *H. pylori* isolated from randomly selected 5 DU patients who had received a gastrointestinal endoscopic examination and 5 GC patients who had received gastrectomy surgery were cultured and total protein extracted, trypsin digested, and labeled with iTRAQ reagents 115 (DU samples) or 117 (GC samples), then the two sets of iTRAQ-labeled peptides were pooled and analyzed on an LTQ-Orbitrap Velos hybrid mass spectrometer. A total of 591 proteins were identified in the combined samples, and, using a p value < 0.05 and a 117/115 ratio > 1.5, 99 proteins were considered statistically reliable hits (Supplemental Table I).

### Pathway analysis and validation of the candidate biomarker proteins

**Figure 2:**
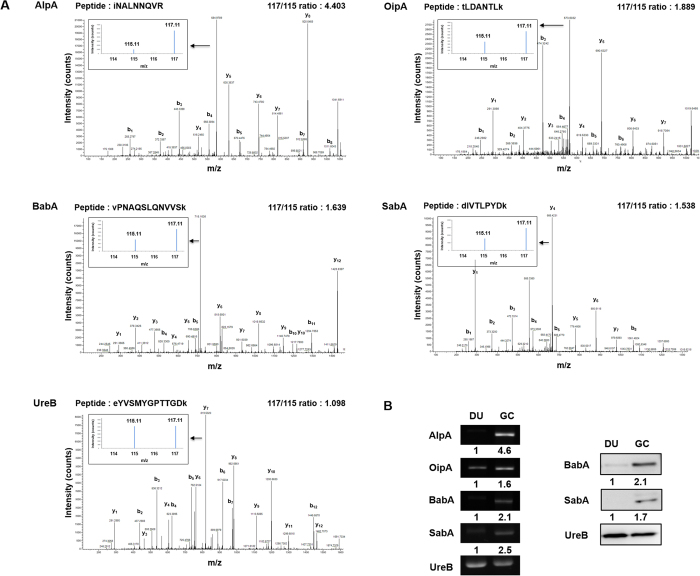
GC-derived *H. pylori* candidate identification and validation. (**A**) The MS spectrum (large panel), iTRAQ spectrum (small panel), identified peptide sequence, and quantified 117/115 iTRAQ (GC/DU) ratio for AlpA, OipA, BabA, SabA, and UreB. (**B**) mRNA and protein extracts from pooled DU-derived or GC-derived *H. pylori* samples (n = 5 per group) were prepared and mRNA levels for AlpA, OipA, BabA, SabA, and UreB (loading control) examined by RT-PCR (left panel) and protein levels of BabA, SabA, and UreB examined by Western blotting (right panel). The data shown are representative of the results obtained in three independent experiments, all gels and blots were run in the same experimental conditions. Uncropped gels and blots are reported in Supplemental Figure 6. The numbers below the panel show the densitometric results for the GC samples normalized to the DU samples.

**Figure 3:**
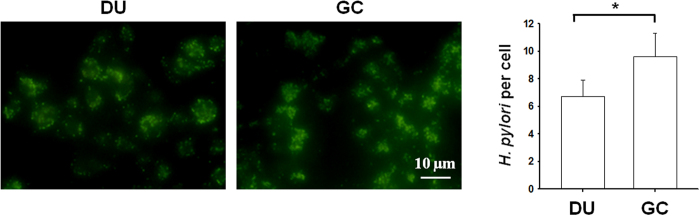
Adherence of DU-derived and GC-derived *H. pylori* to KATO-III cells. KATO-III cells were incubated for 16 h with pooled DU-derived or GC-derived *H. pylori* (n = 5) at an MOI of 10, then were fixed and immunostained with FITC-labeled anti-*H. pylori* antibodies. The left panels show representative images and the right panel shows the quantified results expressed as the mean ± S.D. *p < 0.05. The data shown are representative of those obtained in three independent experiments.

To identify altered biological functions and pathways that might play a role in GC carcinogenesis, we analyzed the 99 proteins showing increased expression (>1.5 fold) in *H. pylori* strains from GC patients compared to DU patients using the Kyoto Encyclopedia of Genes and Genomes (KEGG) pathway database and found that 40 proteins were categorized into the four reaction networks of metabolism, genetic information processing, environmental information processing, and human diseases (Table 1). These pathway results led us to focus on proteins with increased expression that could play a role in causing human diseases (KO pathway: ko05120, epithelial cell signaling in *H. pylori* infection) including AlpA, Cag pathogenicity island proteins Cag1 and CagC, OipA, BabA, and SabA. Cag1 and CagC are involved in the type IV secretion system to inject CagA into gastric epithelial cells, but, since the majority of East Asian *H. pylori* clinical isolates (>98%) are CagA-positive and since less than 20% of patients whose *H. pylori* strains with complete Cag pathogenicity island proteins CagPAI developed GC^30^,^31^, we excluded them from our GC-related candidates and focused on the 4 adhesins, AlpA, OipA, BabA, and SabA. Adhesins facilitate the initial colonization and persistence of *H. pylori* infection and have been associated with gastric diseases that may heighten the risk for developing GC^6^. These four adhesins showing increased expression in GC-related *H. pylori* were therefore selected as biomarker candidates. Figure 2A shows the iTRAQ quantitative MS spectra of representative peptides for AlpA, OipA, BabA, and SabA and for UreB, used as a reference. RT-PCR and immunoblotting were used to validate the quantitative proteomics results, with UreB as internal control. As shown in Fig. 2B (left panel), the RT-PCR data showed that expression of AlpA, OipA, BabA, and SabA was higher in GC strain *H. pylori* than in DU strain *H. pylori.* Since antibodies to BabA and SabA were available, these two adhesins were subjected to immunoblot analysis, and the results (Fig. 2B, right panel) were consistent with the MS-based quantitative and RT-PCR results. To examine differences in adherence of DU and GC strain *H. pylori* to the surface of gastric epithelial cells, KATO-III cells were incubated either with *H. pylori* from 5 DU patients or 5 GC patients, fixed, and incubated with FITC-labeled anti-*H. pylori* antibodies, then the number of adherent *H. pylori* per epithelial cell was assessed by fluorescence microscopy. As shown in Fig. 3, the number of fluorescent *H. pylori* was higher using the GC strain of *H. pylori* than using the DU strain. These data show that the candidate proteins AlpA, OipA, BabA, SabA were more highly expressed in the GC strain of *H. pylori,* and that the GC strain of *H. pylori* showed enhanced adherence to gastric epithelial cells.

### Clinical significance of candidate biomarker protein seroreactivity

**Figure 4:**
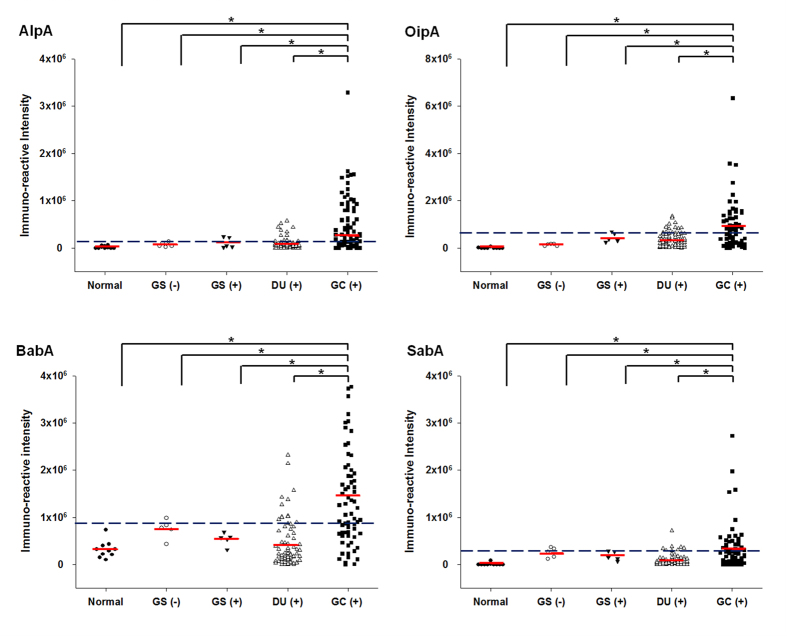
Seroreactivity for AlpA, OipA, BabA, and SabA in normal subjects and patients with gastritis, DU, or GC. Purified recombinant AlpA, OipA, BabA, and SabA were probed with clinical serum samples by Western blotting and the relative immunoreactivity was measured and analyzed. The subjects were healthy individuals without *H. pylori* infection (n = 10), gastritis patients without *H. pylori* infection [GS (−), n = 5], gastritis patients with *H. pylori* infection [GS (+), n = 5], duodenal ulcer patients with *H. pylori* infection [DU (+), n = 80], and gastric cancer patients with *H. pylori* infection [GC (+), n = 76]. The horizontal red lines indicate the mean values for each group and the black dotted line indicates the cut-off value for seropositivity defined as the mean ± 2SD of the non-GC samples. *p < 0.05.

To further examine the clinical significance of AlpA, OipA, BabA, and SabA as potential biomarker candidates for GC-related antigens, the recombinant His-tagged fusion proteins were expressed and purified, and their antigenicity examined by immunoblot analysis using serum from one GC patient, which showed that all 4 recombinant proteins were recognized (Supplemental Fig. 1). Meanwhile, their identity was confirmed by mass spectrometry (data not shown). The reactivity of serum samples with these candidate proteins was then examined by immunoblotting using serum samples from non-*H. pylori-*infected normal individuals (n = 10) or gastritis patients without *H. pylori* infection (n = 5), gastritis patients with *H. pylori* infection (n = 5), DU patients with *H. pylori* infection (n = 80), and GC patients with *H. pylori* infection (n = 76). As shown in Fig. 4, for all 4 antigens, the immunoreactivity of serum samples from GC patients was higher than that in samples from the other groups and the results for more than 46% of the GC patients were above the cutoff value (mean + 2SD for the results for the non-GC patients, i.e. the DU and gastritis patients and normal individuals), suggesting that they are GC-related antigens. As shown in Table 2, the percentage of samples reactive with each of the 4 adhesins was significantly higher than in DU patients or the non-GC group. The prevalence of AlpA seropositivity in patients with GC, DU, non-GC was 46.1, 15, and 14%; which was higher in GC samples than in DU samples (OR = 4.84; 95% CI, 2.26–10.36; p < 0.0001) or the non-GC samples (OR = 5.24; 95% CI, 2.55–10.80; p < 0.0001). OipA seropositivity in GC patients was significantly higher than in DU and the non-GC group (51.3% *versus* 12.5% and 11%), with the highest OR (GC *versus* DU and non-GC; OR = 7.38 and 8.53; 95% CI, 3.31–16.43 and 3.94–18.44; p < 0.0001 and <0.0001) among the 4 antigens. BabA seropositivity in GC patients was significantly higher than in DU patients (53.9% *versus* 13.8%; OR = 7.35; 95% CI, 3.37–16.03; p < 0.0001) or the non-GC group (53.9% *versus* 12%; OR = 8.59; 95% CI, 4.05–18.24; p < 0.0001). The prevalence of SabA seropositivity in GC patients was also significantly higher than in DU patients (48.7% *versus* 12.5%; OR = 6.64; 95% CI, 2.98–14.79; p < 0.0001) or the non-GC groups (48.7% *versus* 15%; OR = 5.38; 95% CI, 2.64–10.93; p < 0.0001). These results show that the prevalence of AlpA, OipA, BabA, and SabA seropositivity was significantly higher in GC patients than in non-GC patients, with a higher OR and p < 0.0001 and these 4 proteins were therefore considered as GC-related antigens.

### Using the three proteins OipA, BabA, and SabA as biomarkers for GC diagnosis

To evaluate whether reactivity with multiple antigens could increase the ability to distinguish GC patients from patients with DU or gastritis or normal individuals, the percentage of serum samples reactive with only one antigen or with two, three, or four antigens was determined and the results are summarized in Table 3. The percentage of serum samples from GC patients that recognized one, two, three, or four of the antigens was, respectively, 25%, 22.4%, 27.6%, and 11.8%, whereas the corresponding percentages were 22.5%, 10%, 3.8% and 0% for samples from DU patients and 22%, 9%, 4%, and 0% for samples from non-GC patients. As a result, the odds ratios increased as the number of antigens recognized increased, going from 1.15 or 1.18 (GC *versus* DU or non-GC; 95% CI, 0.55–2.40 and 0.59–2.39; p = 0.72 and 0.64) for a single antigen recognized to 2.59 or 2.91 (GC *versus* DU or non-GC; 95% CI, 1.05–6.43 and 1.22–6.97; p = 0.03 and 0.01) for two, and to the highest odds ratios 9.8 or 9.16 (GC *versus* DU or non-GC; 95% CI, 2.78–34.49 and 2.99–28.07; p < 0.0001 and <0.0001) for three; the odds ratios for all four antigens were not determined as none of the serum samples from DU or non-GC patients recognized all four antigens.

Gender and age are also the common non-*H. pylori* risk factors for developing GC. GC is more common in men than in women, and the incidence rates rise rapidly after the age of 50 years^32^. The gender and age effects on multiple antigens were then analyzed. As shown in Supplemental Table II, the gender factor does not affect the OR of multiple antigens. In both male and female groups, the OR increased as the number of antigen recognized increased, suggesting the use of multiple antigens can discriminate GC from non-GC patients in both genders. The age effect was also evaluated by dividing patients in two groups by age 50. Similarly, the OR increased as the number of antigen recognized increased in both age under 50 and age over 50 (Supplemental Table III). Notably, the OR for three antigens recognized were higher in the age group over 50, indicating a greater discrimination power of these antigens in elderly population. These results showed that these factors including gender and age may have some impact on the discrimination power of the antigens, but not affect the ability to discriminate GC from non-GC patients.

To further study which three antigens contributed more to the highest odds ratios in the group of three antigens recognized, multiple logistic regression using different combinations of the four antigens as explanatory variables was analyzed. The models indicated that OipA contributes most to the odds, then BabA, then SabA, and the contribution of AlpA was not significant. Using backward/forward stepwise methods, all options from the SPSS software showed that the combination of OipA, BabA, and SabA led to the best prediction model to discriminate GC from the non-GC group [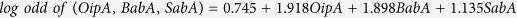, p < 0.01 for all estimated coefficients], with a 77.3% correct prediction rate. These results show that the use of OipA, BabA, and SabA allowed much better discrimination of GC from DU, gastritis, and normal individuals than the use of a single antigen.

### Development of a GC-related protein microarray

**Figure 5:**
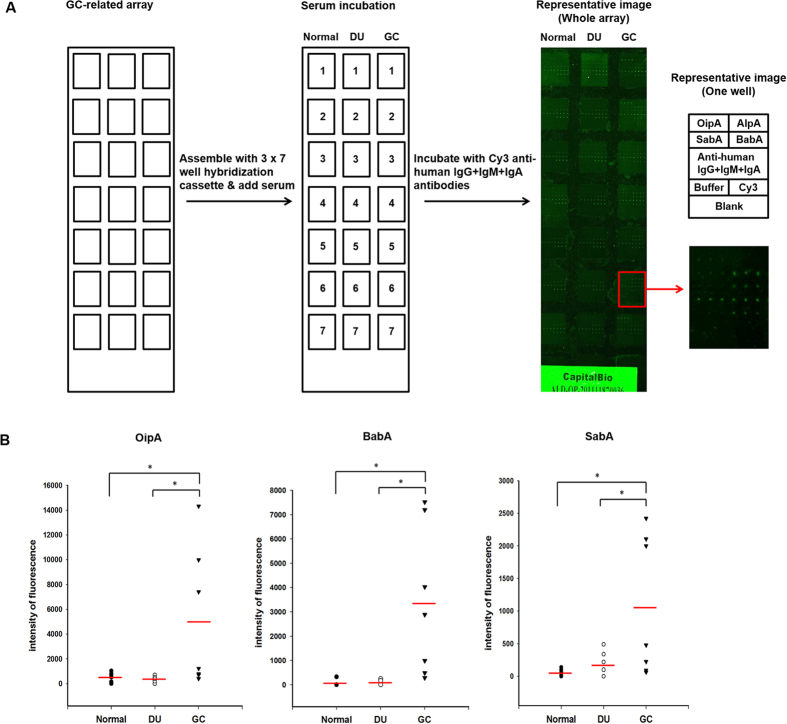
Microarray screening using OipA, BabA, and SabA distinguishes GC patients from DU patients or normal volunteers. (**A**) Left, The selected antigens were purified and printed on a slide for fabrication of the GC-related protein microarray designed for a 3 × 7 well cassette. **Middle**, Individual serum samples from 7 normal individuals, 7 DU patients, and 7 GC patients were applied to individual wells. **Right**, The arrays were then incubated with Cy3-labeled anti-human IgG + IgM + IgA antibodies (green). The left panel shows a representative image of the probed GC-related array, while the panels on the right show a representative well, indicated in red on the left panel; the top panel shows the layout of the well and the bottom panel shows the result. Each antigen was printed in triplicate on the chip. Four concentrations of anti-human IgG + IgM + IgA antibodies (18.75–150 μg/ml) were used as positive controls, buffer was used as a negative control, and Cy3-labeled anti-mouse IgG was used as landmark. (**B**) Comparison of the seroreactivity of the samples from normal individuals, DU patients, or GC patients (n = 7 per group) with the three GC-related antigens, OipA, BabA, and SabA detected as the intensity of fluorescence of the Cy3-labeled anti-human IgG + IgM + IgA antibodies. *p < 0.05.

To improve the efficiency and convenience of diagnosis, a GC-related protein microarray was designed by printing the GC-related antigens on aldehyde slides applying a 3 × 7 well cassette (Fig. 5A). Anti-human IgG, IgM, and IgA antibodies were printed at four serial dilutions (18.75–150 μg/ml) as positive controls and Cy3-labeled anti-mouse IgG was also printed and used for normalization of the intensities in different wells. The GC-related array was then incubated with serum from normal individuals or DU or GC patients (n = 7 per group) in separate wells, then with Cy3-labeled anti-human IgG + IgM + IgA antibodies, and the Cy3 signal at 532 nm was detected and normalized. All samples were printed in triplicate and gave highly reproducible signals, while the blank control gave no signal, and the signals for anti-human IgG, IgM, and IgA increased in a dose-dependent manner. The OipA, BabA, and SabA spots gave a higher signal intensity using serum from GC patients than DU patients or normal individuals, consistent with the immunoblot results. However, only a low signal intensity was seen for AlpA spots, indicated that AlpA was not suitable for aldehyde slide-based microarray screening. Since the contribution of AlpA to the discrimination of GC from non-GC was not significant, AlpA was excluded, and the statistical significance of different signal intensities for the OipA, BabA, and SabA spots between GC and DU patients or normal individuals was analyzed to evaluate the discrimination power of GC-related protein microarray containing these three virulence antigens. Figure 5B shows that the quantified average fluorescence intensity for each of the three antigens probed with GC serum were significantly higher than when serum from normal individuals or DU patients was used. These results demonstrate that diagnostic screening using the three GC-related antigens, OipA, BabA, and SabA, can be performed using a GC-related protein microarray and can distinguish GC patients from DU patients and normal individuals.

## Discussion

Previous studies have shown that subjects with *H. pylori* infection and gastric ulcers are at increased risk of developing GC, whereas very few DU cases develop GC^4^. The virulence factors expressed by *H. pylori* can predict the development of different gastroduodenal diseases^33^. Moreover, the humoral immune response directed against the pathogen is also an important host element that can help to discriminate between diseases of different severity^19^. In the present study, we found that many proteins were differentially expressed by *H. pylori* from GC patients compared to those from DU patients, suggesting that virulence factors may be one of the determinants of the clinical outcome of *H. pylori* infection. Among the proteins that were highly expressed in GC-derived strains, 4 were adhesins categorized as belonging to human disease-related pathways by KEGG. Seroreactivity for these 4 adhesins, AlpA, OipA, BabA, and SabA, was higher in GC patients than in non-GC patients and they were therefore considered as GC-related virulence antigens. The discrimination between GC and non-GC was optimized when OipA, BabA, SabA were used in combination. Furthermore, a GC-related protein microarray with OipA, BabA, and SabA was shown to be a potential convenient diagnostic method with great discrimination power. Our study strongly suggests that these *H. pylori* adhesins are important virulence factors that are able to activate the humoral immune system and may be involved in gastric carcinogenesis.

Adhesins are bacterial cell-surface proteins that help bacteria adhere to host cells^6^. *H. pylori* adhesins are reported to be virulence factors involved in processes during the early and chronic phases of infection for both colonization and pathogenesis^3^. AlpA, OipA, BabA, and SabA bind, respectively, to laminin, EGFR, fucosylated Lewis b blood group antigens, and sialylated carbohydrates on the surface of gastric epithelial cells, and play roles in the bacterial colonization of the gastric mucosa^33^,^34^,^35^. In our study, we found that the number of bacteria attached to each gastric epithelial cell was considerably higher for GC strain *H. pylori,* which shows higher expression of these adhesins, than for DU strain *H. pylori.* The differential expression of these adhesins in GC- and DU-derived strains may result in different adherence ability and lead to gastric carcinogenesis after long-term infection. In the human stomach, adherence has been shown to play a role in the induction of *H. pylori*-associated IL-8 secretion^36^. It is well recognized that the release of proinflammatory cytokines is closely linked to the pathogenesis of *H. pylori*-associated gastric cancer^37^,^38^. Among the adhesins, AlpA, OipA, and BabA are known to induce IL-8 secretion by gastric epithelial cell lines^34^,^39^,^40^,^41^. The role of these adhesins in inflammation shows that they may be important not only in colonization by helping *H. pylori* adhere to host cells, but also in *H. pylori-*associated gastric carcinogenesis by being involved in immune response induction^6^. Some studies have shown that the on-off status of the *oipA* gene is not related to IL-8 expression *in vitro*^40^,^42^, but the different results in different studies may be due to the use of different *H. pylori* strains and the association with other virulence factors.

Several studies have investigated the geographical regions correlation with OipA, BabA, and SabA genes in the clinical *H. pylori* isolates. The presence of OipA gene correlates to the risk of *H. pylori* associated GC in the population of Japan, China, Dutch and Colombian with odds ratio from 2.43 to 5.69^43^, while it has little effect on the Iran population^44^. In addition, the presence of Bab A2 gene is related to the risk of GC in the population of Columbia, Brazil and Munich with OR of from 2.08 to 5.92^45^. Furthermore, the presence of SabA gene is associated with the risk of GC in the population of Columbia with OR of 2.8^46^. Thus, the above three genes may be involved in increasing the risk of *H. pylori* associated GC among different geographical regions. It was therefore assumed that OipA, BabA, and SabA genes also present in virulent *H. pylori* isolates in Taiwan. Therefore, OipA, BabA, and SabA positive *H. pylori* have a strong correlation to the risk of GC development with high OR which accounts for the seropositivity to OipA, BabA, SabA observed in GC patients.

A number of *H. pylori* outer membrane proteins undergo phase variations including OipA, BabA, and SabA^47^,^48^. To evaluate if the genetic background of the *H. pylori* clinical strains randomly selected in this study correlates with their protein expression phenotype, the genotypes of the OipA, BabA, and SabA in the 5 GC strains were determined. As shown in Supplemental Figures 2–4, the sequences of OipA, BabA, and SabA genes in 5 GC strains were listed. Overall, the three antigens of the 5 GC strains are all genetically positive with some strain specific variable mutations which is commonly found in *H. pylori* genes and has long been thought to contribute to host adaption^49^. The protein expression of BabA and SabA of 5 GC strains were also examined with available antibodies as shown in Supplemental Figure 5. All 5 GC strains expressed BabA and SabA protein. These findings implied that as these virulence genes are variable in genetics level, detection of their protein expression status for clinical outcomes correlation should be more reliable.

The variety of pathologies related to *H. pylori* is a consequence of both host and bacterial factors. Besides bacterial virulence factors, host immunological factors, including humoral immune responses directed against bacterial virulence factors, are important^50^. *H. pylori* infection induces inflammation and a strong humoral immune response to a variety of *H. pylori* antigens^51^, and *H. pylori* antigens are therefore regarded as potential candidates for serologic biomarkers^52^. In our study, the adhesins were expressed at higher levels in GC-related *H. pylori*, suggesting that they may cause a higher host response. This suggests that both humoral immune responses and bacterial virulence factors should be taken into account to help in discriminating between diseases of different severity. These GC-related virulence antigens are involved in similar functions^7^,^33^,^35^ and there might have be some interactions between them, and patients recognizing more antigens may have a higher risk of GC. Statistical analysis revealed that reactivity with a greater number of these antigens was related to an increased incidence of GC. The odds ratios for GC *versus* non-GC increased significantly to 9.16 for reactivity with three antigens. Among the different combinations of three of the four antigens, the combination of OipA, BabA, and SabA gave the best prediction model for distinguishing GC patients from DU patients and normal individuals. Heterogeneity of the host immune response to different antigens may affect the occurrence of multiseropositivity in individual patients^53^. Based on our results, we suggest considering seropositivity of three antigens as a high risk of developing GC, and seropositivity of all four antigens should be examined in a larger sample of *H. pylori*-infected patients to verify and to optimize the discrimination of GC from other gastric diseases.

Serum antibodies are elicited by *H. pylori* virulence antigens during infection. The serum antibodies typically become present approximately 21 days after infection and can remain long after eradication. The value of serum antibodies as predictive markers for the gastric diseases remains controversial because serum antibodies are frequently found regardless of the status of gastric diseases^31^. However, previous studies showed that higher *H. pylori* seroprevalence is present in GC patients at early stages of tumor development compared those at advanced stages^54^,^55^. It was also reported that the staging of GC is correlated with the antibody titer of a virulence factor CagA, that the seropositivity of CagA was higher in early stage of GC than in advanced GC, indicating anti-CagA antibody may be used to detect GC in early stage^10^. In our study, we identified OipA, BabA, and SabA can distinguish GC from DU, gastritis and normal individuals. With dividing patients in two groups by age 50, we found that the combination of three markers allowed discrimination of GC from non-GC patients in age under 50, which may present earlier stages of GC carcinogenesis. Even more, the discrimination power became stronger in patients with age over 50, indicating the three antigens may coordinate with the progression of GC. To evaluate whether these markers can be used to detect early stage GC diagnosis, a change of antibody titers in serum of these markers should be determined in the future.

To apply multiple biomarkers for GC diagnosis, protein microarray technology was used^56^. A protein microarray containing the GC-related antigens was fabricated and the antibody binding patterns of serum samples from normal individuals, DU patients, or GC patients were compared. The signal intensities for the OipA, BabA, and SabA spots were higher using serum samples from GC patients than using samples from DU patients or normal subjects, but no signals were seen using AlpA. Comparing the two techniques used in this study, Western blotting is suitable for antigen screening but needs more serum for multiple antigens; whereas protein microarray can quantify multiple antigens in parallel using the same amount of serum, but the immobilization of different antigens on the chip may vary^57^,^58^. To overcome this problem, it would be necessary to develop efficient immobilizing techniques which increase binding of antigens. However, even without AlpA, the GC-related protein microarray containing three antigens proved the potential of GC antigen microarray as a clinical diagnostic platform.

These findings not only provide a better biomarker that can distinguish GC from DU for clinical use, but also indicate that GC-related strain *H. pylori*, with higher expression of these adhesins, may have other effects on host cells involved in gastric carcinogenesis. In conclusion, we found that the four adhesins AlpA, OipA, BabA, and SabA are GC-related virulence antigens. Combination of OipA, BabA, and SabA provides a good discrimination for GC screening, and a GC-related protein microarray containing these three antigens could provide a novel, rapid, and convenient platform for clinical high-throughput *H. pylori-*associated GC screening.

## Methods

### Bacterial strains and culture conditions

Clinical *H. pylori* strains were isolated from endoscopic biopsy specimens from the stomachs of patients with DU or GC at the National Taiwan University Hospital. The different strains were used to inoculate Columbia agar containing 5% sheep blood (Invitrogen, Grand Island, NY) and were grown at 37 °C in a microaerophilic chamber (Don Whitley, West Yorkshire, UK) in 10% CO_2_, 5% O_2_, and 85% N_2_.

### Patients and serum samples

Both serum samples and the related gastric biopsy specimens were prospectively collected from individuals in the National Taiwan University Hospital with patients’ informed consent and these procedures were approved by the Research Ethics Committee of National Taiwan University. Eighty patients with DU and 10 patients with gastritis received an upper gastrointestinal endoscopic examination and 76 patients with GC underwent curative intent radical were enrolled. *H. pylori* status was determined by culture and/or histological examination of gastric biopsy specimens. GC patients were staged according to the World Health Organization and Lauren’s classification^59^. The clinical features of GC patients are listed in Supplemental Table IV. In addition, 10 individuals with a minimal histological change of the gastric mucosa and no evidence of *H. pylori* infection were selected as controls. The procedures were carried out in accordance with the guidelines approved for research on human samples.

### Sample preparation for iTRAQ analysis

*H. pylori* cells from randomly selected 5 DU or 5 GC patients were lysed by sequential passage through a French Press Cell (Thermo Scientific, Waltham, MA) in lysis buffer (7 M urea, 2 M thiourea, 4% CHAPS, pH 8.5). The lysate of the combined *H. pylori* samples from 5 DU patients or 5 GC patients containing 100 μg of protein was subjected to iTRAQ analysis following the manufacturer instructions (SCIEX, Framingham, MA). The protein mixtures were dissolved, reduced, alkylated, and digested overnight at 37 °C in trypsin solution (trypsin:protein ratio 1:50), then the peptides produced from the *H. pylori* cells from DU patients or GC patients were labeled, respectively, with iTRAQ reagents. Three iTRAQ analyses using three independently isolated *H. pylori* protein samples from DU and GC patients were performed.

### Off-line 2D-LC-MS/MS

Equal amounts (100 μg) of the iTRAQ-labeled 115 DU peptides and the 117 GC peptides were mixed, concentrated and desalted using a C18 cartridge. The peptides were resuspended with 2% acetonitrile and 0.1% formic acid and were trapped on a reverse phase C18 column (Acclaim PepMap100, 3 μm, 100 Å, 75 μm × 2 cm; Dionex, Sunnyvale) and separated using coupled reverse phase C18 chromatography (Acclaim PepMap RSLC, 2 μm, 100 Å, 75 μm × 15 cm; Thermo Scientific) with an acetonitrile gradient in 0.1% formic acid. The injection volume was 2 μl, and the peptides were then separated by gradient elution at 250 nL/min in 90 min. Full-scan MS spectra (m/z 300–1600) were acquired in an Orbitrap mass analyzer (Thermo Scientific) at a resolution of 60,000. The lock mass was set at 445.12003 (polycyclodimethylsiloxane, PCM).

### Protein identification and quantification

The precursor mass tolerance was set at 7 ppm and the fragment ion mass tolerance at 0.5 Da. The dynamic modifications were deamidated (NQ), oxidation (M), and N-terminal acetylation. The static modification was cysteine carbamidomethylation, and a maximum of two miscleavages were allowed. Identified peptides were validated using a Percolator algorithm with a q-value threshold of 0.01. Mass spectrometry data were processed and quantified using Proteome Discoverer version 1.3 (Thermo Scientific) and the Mascot search engine (version 2.3.02) to search the NCBI *H. pylori* protein database. The search parameters were set using methyl methanethiosulfonate as cysteine, iTRAQ 4-plex at lysine, and the N-terminal residue as static modifications. Fragment ion mass tolerance and precursor ion tolerance were set to 0.2 Da with a 95% confidence threshold.

### RT-PCR

*H. pylori* cells from DU or GC patients (n = 5 per group) were collected and mRNA isolated using RNAspin mini RNA isolation kits (GE Healthcare, Kowloon, HK). Reverse transcription reactions were performed according to the instruction manual for SuperScriptTM First-Strand Synthesis System for RT-PCR (Invitrogen). The resulting cDNA was used as template for PCR amplification of AlpA, OipA, BabA, SabA, and urease subunit B (UreB, loading control) using the primer pairs listed in Supplemental Table V.

### Immunoblot analysis

Proteins extracts of *H. pylori* (20 μg /lane) were separated by SDS-PAGE and transferred to a PVDF membrane. After blocking by 5% nonfat dry milk in TBS (pH 7.5) for 1 h at 25 °C, the blot was incubated overnight at 4 °C with anti-BabA, SabA antibodies (1:2000; provided by Dr. Jiunn-Jong Wu) or anti-UreB antibodies (1:500, Abcam, Hong Kong, China) as loading control. After 6 washing steps, the membranes were incubated with HRP-labeled anti-rabbit IgG antibodies (Santa Cruz, Santa Cruz, CA). After another 6 six washing steps, bound antibodies were detected using ECL reagents, visualized on an LAS4000 Luminescent Image Analyzer (Fujifilm, Tokyo, Japan), and quantified using MultiGauge software (Fujifilm).

### Adherence assay

The adherence assay was performed as described previously^60^. KATO-III gastric epithelial cells were seeded on coverslips overnight, incubated for 16 h with DU or GC strain *H. pylori* (n = 5) at an MOI = 10, fixed with 4% paraformaldehyde in PBS for 15 min at room temperature, and blocked by incubation for 1 h at room temperature with 5% BSA. They were then immunostained for 1 h at room temperature with FITC-labeled rabbit antibodies against *H. pylori* (Abcam; 1:200 in PBS), and images were captured using a BX51 fluorescence microscope (Olympus, Tokyo, Japan). The results were expressed as the number of fluorescent *H. pylori* per cell calculated from fluorescence frequency distribution histograms.

### Cloning and purification of recombinant proteins

*H. pylori* genomic DNA was extracted using DNA purification kits (Qiagen, Venlo, Netherlands) and the genes encoding AlpA, OipA, BabA, and SabA were cloned using the expression vector pET28a (Novagene, Bedford, MA); the sequences of the primers used are listed in Supplemental Table V. Expression of each protein, bearing a 6-His tag, was induced in *E. coli* strain BL21 by incubation for 4 h at 37 °C with 1 mM isopropyl β-D-1-thiogalactopyranoside (Sigma, St. Louis, MO), then the recombinant proteins were dissolved in binding buffer (20 mM Tris-HCl, 0.5 M NaCl, 5 mM imidazole, pH 7.9, containing 8 M urea) and purified on a Ni^2+^-chelating Sepharose column (GE Healthcare) by elution with binding buffer containing 200 mM imidazole. All proteins were >95% pure, as shown by Coomassie Blue staining of SDS-PAGE gels.

### Serologic study

Recombinant AlpA, OipA, BabA, and SabA were electrophoresed and transferred. The PVDF membrane was incubated for 1 h at room temperature with blocking buffer, and then incubated overnight at 4 °C with serum samples from normal individuals or gastric disease patients diluted 1:500 in blocking buffer, then, after 6 washes with TBST, was incubated for 1 h at 25 °C with HRP-conjugated goat anti-human IgG antibody (Jackson Immunoresearch Laboratories, West Grove, PA) diluted 1:2000 in blocking buffer. After another 6 TBST washes, bound antibodies were detected using ECL reaction solution, visualized on an LAS4000 and quantified using MultiGauge software.

### Statistical analysis

All experiments were performed three times and the data are expressed as mean ± SD. Student’s t test was used to determine the significance of differences between serum reactions, with a p value < 0.05 being considered statistically significant. The odds ratio (OR) and 95% confidence interval (CI) were determined using MedCalc version 12.7. Differences between the reactivity of serum from normal individuals or patients were analyzed using the Chi-squared test or Fisher’s exact test. Multiple logistic regression analysis was used to estimate the prediction model with multiple antigens by SPSS version 17.0.

### Protein microarray fabrication

The purified recombinant proteins were printed at 20 °C on an aldehyde slide using a SmartArrayer 136 (CapitalBio, Beijing, China) to fabricate a GC-related protein microarray using a protocol modified from a previous study^56^. The chips were kept at 20 °C for protein immobilization for at least 8 h. The format of the array was designed for a 3 × 7 well cassette. Each array contained different concentrations of anti-human IgG + IgM + IgA antibodies (18.75–150 μg/ml, Jackson Immunoresearch Laboratories) as controls, Cy3-labeled anti-mouse IgG antibodies (Jackson Immunoresearch Laboratories) as landmark, and buffer as a negative control.

### Serum reactions on protein microarrays

The protein microarray was blocked for 1 h at room temperature with 3% BSA in TBST. After 3 TBST washes, the array was assembled using a 3 × 7 well hybridization cassette (Arrayit Corporation, Sunnyvale, CA) and incubated sequentially in individual wells for 1 h at room temperature with serum (1:100 in 1% BSA in TBST), then, after 3 TBST washes, with Cy3-conjugated rabbit anti-human IgG + IgM + IgA antibodies (1:20000 in 1% BSA in TBST, Jackson Immunoresearch Laboratories). The array was washed with TBST and distilled water, dried by centrifugation on a SlideWasher (CapitalBio), and imaged using a LuxScan scanner (CapitalBio) with an excitation wavelength of 532 nm and emission wavelength of 570 nm, then image analysis was performed using GenePix Pro 6.0 (Axon Instruments, Sunnyvale, CA).

## Additional Information

**How to cite this article**: Su, Y.-L. *et al*. Combination of OipA, BabA, and SabA as candidate biomarkers for predicting *Helicobacter pylori*-related gastric cancer. *Sci. Rep.* **6**, 36442; doi: 10.1038/srep36442 (2016).

**Publisher’s note**: Springer Nature remains neutral with regard to jurisdictional claims in published maps and institutional affiliations.

## Supplementary Material

Supplementary Information

## Figures and Tables

**Table 1:** Proteins upregulated (fold change > 1.5) in *H. pylori* strains from GC patients categorized by KEGG pathway.

KEGG pathway	Protein	Number of proteins
Metabolism	SerC, HyuA, PetC, OorD, Ppa, CA, FixO, AspA, AtpF, Ggt, RpoZ, AroD, AckA, Asd, PdxJ, PetA, FabZ, AtpD, KdsA	19
Genetic information processing	RplC, DnaB, RplM, RpoZ, GroEL, RpsQ, Ffh, RpsC, RpsJ, RplW, RplU, RpsG, RpsI, RpsB	14
Environmental information processing	PetC, FixO,CeuE Ffh, HtrA, PetA	6
Human diseases	AlpA, Cag1, OipA, CagC, BabA, SabA	6

**Table 2:** Seropositivity with the antigens in patients with gastroduodenal diseases or normal volunteers and the odds ratio.

Antigen	Number positive (%)			OR (95% CI), *p* value^b^	
	GC (n = 76)	DU (n = 80)	non-GC^a^ (n = 100)	GC *vs.* DU	GC *vs.* non-GC
AlpA	35 (46.1)	12 (15)	14 (14)	4.84 (2.26–10.36), <0.0001	5.24 (2.55–10.80), <0.0001
OipA	39 (51.3)	10 (12.5)	11 (11)	7.38 (3.31–16.43), <0.0001	8.53 (3.94–18.44), <0.0001
BabA	41 (53.9)	11 (13.8)	12 (12)	7.35 (3.37–16.03), <0.0001	8.59 (4.05–18.24), <0.0001
SabA	37 (48.7)	10 (12.5)	15 (15)	6.64 (2.98–14.79), <0.0001	5.38 (2.64–10.93), <0.0001

^a^The non-GC group consisted of normal subjects, gastritis patients with or without *H. pylori* infection, and DU patients.

^b^*p* value calculated using the Chi-squared test.

**Table 3:** Effect of an increased number of recognized antigens on the odds ratio.

No.	Recognized antigens	Number positive (%)			OR (95% CI), *p* value^b^	
	Antigens	GC (n = 76)	DU (n = 80)	non-GC^a^ (n = 100)	GC *vs.* DU	GC *vs.* non-GC
0	None	10 (13.2)	51 (63.8)	65 (65.0)	0.09 (0.04–0.20), <0.0001	0.08 (0.04–0.18), <0.0001
1	Any one antigen	19 (25)	18 (22.5)	22 (22.0)	1.15 (0.55–2.40), 0.72	1.18 (0.59–2.39), 0.64
2	Two of the four antigens	17 (22.4)	8 (10)	9 (9.0)	2.59 (1.05–6.43), 0.03	2.91 (1.22–6.97), 0.01
3	Three of the four antigens	21 (27.6)	3 (3.8)	4 (4.0)	9.8 (2.78–34.49), <0.0001	9.16 (2.99–28.07), <0.0001
4	All four antigens	9 (11.8)	0 (0)	0 (0)	ND^c^	ND

^a^The non-GC group consisted of normal subjects, gastritis patients with or without *H. pylori* infection, and DU patients.

^b^*p* value calculated using the Chi-squared test.

^c^ND, not determined.
