# Supplementary material for: Combination of OipA, BabA, and SabA as candidate biomarkers for predicting Helicobacter pylori-related gastric cancer
Source: Sci Rep. 2016 Nov 7;6:36442. doi: 10.1038/srep36442 (PMC5098209; doi:10.1038/srep36442)
Supplement: Supplementary Information [file srep36442-s1.pdf]

Combination of OipA, BabA, and SabA as candidate biomarkers for predicting *Helicobacter pylori*-related gastric cancer

Yu-Lin Su[1], Hsiang-Ling Huang[1], Bo-Shih Huang[1], Po-Chung Chen[2], Chien-Sheng Chen[2], Hong-Long Wang[3], Pin-Hsin Lin[1], Meng-Shu Chieh[4], Jiunn-Jong Wu[5], Jyh-Chin Yang[6*], and Lu-Ping Chow[1,7*]

[1]Graduate Institute of Biochemistry and Molecular Biology, College of Medicine, National Taiwan University, Taipei, Taiwan; [2]Graduate Institute of Systems Biology and Bioinformatics, National Central University, Taoyuan, Taiwan; [3]Department of Statistics, National Taipei University, New Taipei City, Taiwan; [4]First Core Laboratory, College of Medicine, National Taiwan University, Taipei, Taiwan; [5]Department of Medical Laboratory Science and Biotechnology, Center of Infectious Disease and Signaling Research, College of Medicine, National Cheng Kung University, Tainan, Taiwan; [6]Department of Internal Medicine, Hospital and College of Medicine, National Taiwan University, Taipei, Taiwan; [7]Center of Genomic Medicine, National Taiwan University, Taipei, Taiwan.

**Address correspondence to Dr. Lu-Ping Chow, Graduate Institute of Biochemistry and Molecular Biology, College of Medicine, National Taiwan University, No. 1, Sec. 1, Jen-Ai Rd., Taipei 100, Taiwan. Tel: +(886)-2-23123456, ext. 88214. Fax: +(886)-2-23958814. E-mail address: chowip@ntu.edu.tw or Dr. Jyh-Chin Yang, Department of Internal Medicine, Hospital and College of Medicine, National Taiwan University, No. 7, Chung-Shan South Rd., Taipei 100, Taiwan. Tel: +(886)-2-23123456, ext.65055. E-mail: jcyang47@ntu.edu.tw.

Supplemental Method

Molecular genotyping by sequencing

The genomic DNA of GC *H. pylori* stains (n=5) were extracted and OipA, BabA and SabA genes were amplified by PCR as templates for cycle sequencing with BigDye® Terminator v3.1 Cycle Sequencing Kit (Thermo Fisher Scientific). The extension products were analyzed by capillary electrophoresis using an ABI 3730 x1 DNA sequencer (Applied Biosystems, Foster City, CA). The primers used for sequencing are listed in Supplemental Table V. The nucleotide sequences were analyzed by ApE version 2.0 and BLAST system at the NCBI.

Supplemental Table I. Proteins upregulated (fold change > 1.5) in *H. pylori* strains from GC patients compared to DU patients

Accession number	Protein name[a]	Mascot score	Coverage (%)	Matched peptides	117/115 ratio[b]
118596700	**Adherence-associated lipoprotein A (AlpA)**	74.44	10.64	6	4.403
188144165	Hypothetical protein HPSH_05890	69.15	2.78	1	3.902
325997176	Acylamide aminohydrolase	52.47	10.70	4	2.905
107837644	Outer membrane protein (HorJ)	45.84	8.47	3	2.766
317182137	Thioredoxin	437.38	37.74	4	2.656
147667712	**Cag pathogenicity island protein 1 (Cag1)**	89.65	21.74	2	2.525
207108417	Hypothetical protein HpylH_02130	50.22	8.43	2	2.399
15611194	Bacterioferritin comigratory protein	50.24	7.24	1	2.379
207110092	Flagellar assembly protein (FliW)	45.18	26.92	1	2.284
317014107	**N-methylhydantoinase (SerC)**	374.00	15.95	17	2.208
166865295	Cytotoxin associated protain A	121.76	25.00	3	2.165
297379894	Hydantoin utilization protein B (HyuB)	459.10	18.95	20	2.149
315586631	**Hydantoin utilization protein A (HyuA)**	880.87	18.37	13	2.123
207093328	Hypothetical protein HpylHP_11291	139.19	25.39	5	2.047
317010246	**Ubiquinol cytochrome c oxidoreductase, cytochrome c1 (PetC)**	82.32	17.89	5	2.042
315113175	Chain A, solution structure of Slyd	154.74	16.56	3	2.021
15645213	**2-oxoglutarate:acceptor oxidoreductase (OorD)**	80.50	13.27	1	2.008
317181408	Hypothetical protein HPF57_0118	73.89	7.04	4	1.983
15611698	Hypothetical protein jhp0631	148.11	25.90	6	1.921
15612304	**50S ribosomal protein L3 (RplC)**	138.07	26.18	6	1.905
15611609	Hypothetical protein jhp0542	52.97	7.35	4	1.898
315586681	**Outer inflammatory protein (OipA)**	40.07	5.92	2	1.890
188144314	Conserved hypothetical secreted protein	357.59	39.01	10	1.873

317182005	Acetyl coenzyme A acetyltransferase	136.26	20.20	6	1.850
107836198	Hypothetical protein HPAG1_1536	323.62	33.60	10	1.849
317182811	Hypothetical protein HPF57_1521	303.84	33.60	10	1.849
25808535	**Inorganic pyrophosphatase (Ppa)**	45.53	10.40	3	1.847
207092998	**Carbonic anhydrase (CA)**	25.02	4.96	2	1.846
15646105	Unnamed protein product	84.14	26.40	5	1.846
297379391	Response regulator (OmpR)	76.91	20.00	5	1.845
188143179	Hypothetical protein HPSH_00680	69.04	50.00	2	1.844
317182706	Putative outer membrane protein	58.51	4.04	2	1.839
317181400	Hypothetical protein HPF57_0110	88.91	41.27	3	1.817
317015027	Hypothetical protein HPGAM_08495	280.74	30.83	9	1.798
95117797	Outer membrane protein (Omp22)	550.30	35.20	7	1.768
15645472	Unnamed protein product	31.50	3.00	2	1.765
317012238	Translation initiation factor (IF-2)	117.26	10.36	10	1.740
207109410	**Cytochrome c oxidase cbb3-type subunit II (FixO)**	79.90	22.66	4	1.736
46251334	Outer membrane protein	339.21	33.33	5	1.730
127663933	**Cag pathogenicity island protein 25 (CagC)**	42.07	21.74	3	1.727
107837310	Hypothetical protein HPAG1_1112	192.41	19.67	3	1.717
107836832	**Aspartate ammonia-lyase (AspA)**	156.98	13.89	8	1.712
217033713	Hypothetical protein HP9810_5g55	36.36	5.73	2	1.704
207109532	**Replicative DNA helicase (DnaB)**	38.18	5.59	1	1.695
325996403	**ATP synthase F0F1 subunit B (AtpF)**	56.13	17.91	3	1.690
107837708	**Iron(III) ABC transporter, periplasmic iron-binding protein (CeuE)**	178.01	19.70	8	1.683
307637404	Hypothetical protein hp908_0728	122.25	14.38	2	1.683
207108785	**50S ribosomal protein L13 (RplM)**	53.39	23.53	5	1.683
11321265	**Gamma-glutamyltranspeptidase (Ggt)**	84.29	9.17	6	1.681

207109162	**DNA-directed RNA polymerase subunit omega (RpoZ)**	42.47	32.88	3	1.676
188144468	Hypothetical protein HPSH_07460	249.33	16.19	3	1.675
15611420	Copper-associated protein	70.71	16.67	1	1.675
317010684	**3-dehydroquinate dehydratase (AroD)**	62.57	10.78	1	1.674
110377354	**Heat shock protein 60 (GroEL)**	3425.88	77.11	42	1.665
208435298	ATP/GTP binding protein	138.71	4.35	2	1.659
317177289	Outer membrane protein (HofC)	357.43	14.77	8	1.657
207092727	ATP-binding protein (Mpr)	38.35	21.32	3	1.655
15644733	Unnamed protein product	416.82	18.76	10	1.653
107837702	Translation elongation factor (EF-Ts)	231.96	28.17	12	1.650
188143969	**Acetate kinase (AckA)**	36.68	5.24	4	1.646
712831	Heat shock protein	2499.12	76.15	40	1.635
188144216	**Aspartate-semialdehyde dehydrogenase (Asd)**	66.17	7.23	3	1.630
217034805	Hypothetical protein HP9810_866g2	360.08	40.13	7	1.622
317013097	**30S ribosomal protein S17 (RpsQ)**	68.83	24.42	4	1.621
15612352	Rod shape-determining protein (MreB)	129.06	11.82	3	1.615
317181264	Hypothetical protein HPF32_1468	188.68	25.23	11	1.612
317011348	**Signal recognition particle protein (Ffh)**	83.67	6.92	3	1.608
317008739	Urease accessory protein (UreH)	42.01	7.92	2	1.603
15644849	Unnamed protein product	73.05	15.95	6	1.603
207109368	Biopolymer transport protein	42.87	8.18	1	1.597
315587202	**30S ribosomal protein S3 (RpsC)**	148.34	33.76	13	1.593
207108543	**Pyridoxal phosphate biosynthetic protein (PdxJ)**	55.12	8.85	3	1.590
107836475	ATP-dependent nuclease	28.68	8.14	4	1.588
294662397	Chain B, crystal structure of Ycei (Hp1286)	250.52	36.59	9	1.587
308062217	**Blood group antigen binding adhesin (BabA)**	414.23	9.62	7	1.586

210135477	**30S ribosomal protein S10 (RpsJ)**	59.24	13.46	2	1.583
15611308	Outer membrane protein	37.53	11.25	7	1.582
317013104	**50S ribosomal protein L23 (RplW)**	253.69	49.46	5	1.573
217034319	Hypothetical protein HP9810_491g12	154.44	29.52	6	1.570
107836496	**50S ribosomal protein L21 (RplU)**	128.73	20.19	3	1.556
15645018	Unnamed protein product	158.50	29.52	6	1.556
15611904	Hydrogenase expression/formation protein	88.21	10.33	5	1.555
315586121	Conserved hypothetical protein	87.74	13.14	4	1.552
15611134	Urease accessory protein	129.96	23.62	5	1.550
207093235	**30S ribosomal protein S7 (RpsG)**	217.42	32.03	6	1.550
148717246	**Sialic acid-binding adhesin (SabA)**	35.62	2.15	2	1.538
188143129	**30S ribosomal protein S9 (RpsI)**	239.38	37.98	6	1.537
15645683	Unnamed protein product	56.31	8.86	7	1.536
188143477	**Serine protease DO (HtrA)**	544.56	30.04	14	1.535
317010118	Ferrodoxin-like protein	38.57	4.37	2	1.535
217034426	Hypothetical protein HP9810_11g8	87.29	9.25	6	1.533
107837687	**Ubiquinol cytochrome c oxidoreductase, 2Fe-2S subunit (PetA)**	69.89	10.18	2	1.533
15644814	Unnamed protein product	92.97	17.98	5	1.530
189011844	Heat shock protein	241.29	53.93	6	1.521
15645986	**(3R)-hydroxymyristoyl-ACP dehydratase (FabZ)**	55.73	16.35	3	1.519
317014518	**F0F1 ATP synthase subunit beta (AtpD)**	574.90	22.60	10	1.516
207108692	**2-dehydro-3-deoxyphosphooctonate aldolase (KdsA)**	87.11	9.55	2	1.515
298736833	Bifunctional methionine sulfoxide reductase A/B	152.90	18.82	9	1.504
15646161	**30S ribosomal protein S2 (RpsB)**	109.01	18.18	7	1.500

[a] Proteins that were categorized by KEGG pathway analysis are shown in bold.

[b] The 117/115 iTRAQ ratios represent the results for three independent repeats.

Supplemental Table II Gender effect on the odds ratio of an increased number of recognized antigens

Recognized antigens		Number positive (%)			OR (95% CI), *p* value[b]	
**Male**	**Antigens**	**GC (n=48)**	**DU (n=56)**	**non-GC[a] (n=69)**	**GC *vs.* DU**	**GC *vs.* non-GC**
	None	5 (10.4)	34 (60.7)	43 (62.3)	0.08 (0.03- 0.22), <0.0001	0.07 (0.02-0.20), <0.0001
	Any one antigen	14 (29.2)	15 (26.8)	18 (26.1)	1.13 (0.48-2.66), 0.79	1.17 (0.51-2.65), 0.71
	Two of the four antigens	12 (25.0)	6 (10.7)	6 (8.7)	2.78 (0.95-8.09), 0.05	3.5 (1.21-10.12), 0.02
	Three of the four antigens	10 (20.8)	1 (1.8)	2 (2.9)	14.47 (1.78-117.82), 0.0016	8.82 (1.84-42.35), 0.0022
	All four antigens	7 (14.6)	0 (0)	0 (0)	ND[c]	ND
**Female**	**Antigens**	**GC (n=28)**	**DU (n=24)**	**non-GC (n=31)**	**GC *vs.* DU**	**GC *vs.* non-GC**
	None	5 (17.9)	17 (70.8)	22 (71.0)	0.09 (0.02-0.33), 0.0001	0.09 (0.03-0.31), <0.0001
	Any one antigen	5 (17.9)	3 (12.5)	4 (12.9)	1.52 (0.32-7.16), 0.71	1.47 (0.35-6.12), 0.72
	Two of the four antigens	5 (17.9)	2 (8.3)	3 (9.7)	2.39 (0.42-13.64), 0.43	2.03 (0.44-9.41), 0.46
	Three of the four antigens	11 (39.3)	2 (8.3)	2 (6.5)	7.12 (1.39-36.48), 0.01	9.38 (1.85-47.47), 0.0024
	All four antigens	2 (7.1)	0 (0)	0 (0)	ND	ND

[a]The non-GC group consisted of normal subjects, gastritis patients with or without *H. pylori* infection, and DU patients.

[b]*p* value calculated using the Chi-squared test.

[c]ND, not determined.

Supplemental Table III. Age effect on the odds ratio of an increased number of recognized antigens

Recognized antigens		Number positive (%)			OR (95% CI), *p* value[b]	
<50	Antigens	GC (n=11)	DU (n=31)	non-GC[a] (n=48)	GC *vs.* DU	GC *vs.* non-GC
	None	1 (9.1)	17 (54.8)	29 (60.4)	0.08 (0.01-0.72), 0.01	0.07 (0.01-0.55), 0.0021
	Any one antigen	4 (36.4)	9 (29.0)	13 (27.1)	1.40 (0.33-5.97), 0.71	1.54 (0.39-6.14), 0.71
	Two of the four antigens	2 (18.2)	3 (9.7)	3 (6.3)	2.07 (0.30-14.44), 0.59	3.33 (0.49-22.90), 0.23
	Three of the four antigens	3 (27.3)	2 (6.5)	3 (6.3)	5.44 (0.77-38.33), 0.10	5.63 (0.96-32.97), 0.07
	All four antigens	1 (9.1)	0 (0.0)	0 (0)	ND[c]	ND
≥50		GC (n=65)	DU (n=49)	non-GC (n=52)	GC *vs.* DU	GC *vs.* non-GC
	None	9 (13.8)	34 (69.4)	36 (69.2)	0.07 (0.03-0.18), <0.0001	0.07 (0.03-0.18), <0.0001
	Any one antigen	15 (23.1)	9 (18.4)	9 (17.3)	1.33 (0.53-3.36), 0.54	1.43 (0.57-3.60), 0.44
	Two of the four antigens	15 (23.1)	5 (10.2)	6 (11.5)	2.64 (0.89-7.85), 0.07	2.3 (0.82-6.43), 0.11
	Three of the four antigens	18 (27.7)	1 (2.0)	1 (1.9)	18.38 (2.36-143.30), 0.0003	19.53 (2.51-152.08), 0.0002
	All four antigens	8 (12.3)	0 (0)	0 (0)	ND	ND

[a]The non-GC group consisted of normal subjects, gastritis patients with or without *H. pylori* infection, and DU patients.

[b]*p* value calculated using the Chi-squared test.

[c]ND, not determined.

Supplemental Table IV. Clinical features of the gastric cancer patients

Feature		No. (%)
**Total**		**76 (100)**
**Gender**		
	**Male**	**48 (63)**
	**Female**	**28 (37)**
**Age (yr)**		
	**Average**	**64.2**
	**Range**	**23-87**
	**SD**	**13.3**
**Staging**		
	**EGC**[a]	**23 (30)**
	**AGC**[b]	**53 (70)**
**Histological type**		
	**Diffuse**	**27 (36)**
	**Non-diffuse**	**49 (64)**
**Tumor location**		
	**Antrum**	**43 (57)**
	**Non-antrum**	**33 (43)**

[a]Early gastric cancer with cancer cell invasion confined to the mucosa or submucosa.

[b]Advanced gastric cancer with cancer cell invasion beyond the muscularis propria.

Supplemental Table V. Primer sequences used for constructs, RT-PCR, and genotyping

Gene	Primer sequence (F : forward, R : reverse)
AlpA	F : 5'-GGATCCGAATCCTGCGCTAGCATAAGTTA-3'
	R : 5'-CTCGAGCTCGAGGAATGAATACCCATAAGA-3'
OipA	F : 5'-GGATCCGAATCCCACGCTGAAAGG-3'
	R : 5'-CTCGAGCTCGAGATAAACGCTCAC-3'
	F1: 5'-CATGCTGAAAGGAACGGATTTTAT-3'
	R1: 5'-ATAAACGCTCACCACTCTTTTATA-3'
BabA	F : 5'-GGATCCGAATCCGAAGACGACGGCTTTTACATGAGC-3'
	R : 5'-CTCGAGCTCGAGAAATACACGCTATAGAGCCTGCGG-3'
	F1: 5'-GAAGACGACGGCTTTTACATGAGC-3'
	R1: 5'-AAATACACGCTATAGAGCCTGCGG-3'
	F2: 5'-CGGGACGGTGAATGTAACCT-3'
	R2: 5'-ATTTTCCTGAAGGGGTTACG-3'
SabA	F : 5'-GGATCCGAATCCGAAGACAACGGCTTTTTTGT-3'
	R : 5'-CTCGAGCTCGAGTTAATAAGCAAACACATAATTGAG-3'
	F1: 5'-GAAGACAACGGCTTTTTTGT-3'
	R1: 5'-TTAATAAGCAAACACATAATTGAG-3'
	F2: 5'-TGCTCAGGAATTGAAAAC TG-3'
	R2: 5'-CCGTTCAAAGCGCCGTTATTGTT-3'
UreB	F : 5'-ATGAAAAAGATTAGCAGAAAAGAATAT-3'
	R : 5'-CTAGAAAATGCTAAAGAGTTGCGCCA-3'

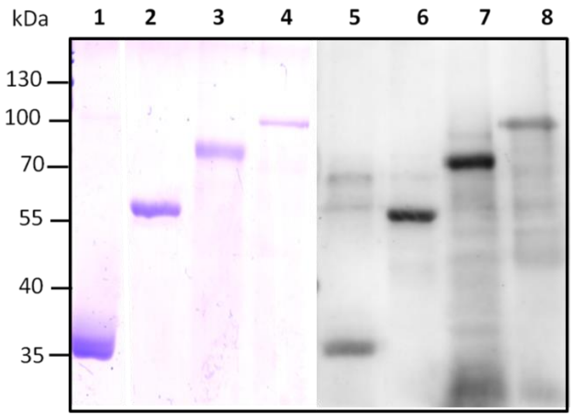

Supplemental Figure 1. Purity of the recombinant proteins and reactivity with a GC patient serum. Purified recombinant OipA (lanes 1 and 5), AlpA (lanes 2 and 6), SabA (lanes 3 and 7), and BabA (lanes 4 and 8) were electrophoresed on a 10 % SDS-PAGE and either stained with Coomassie Blue (lanes 1-4) or transferred to a PVDF membrane and immunoblotted with serum from a GC patient serum (lanes 5-8).

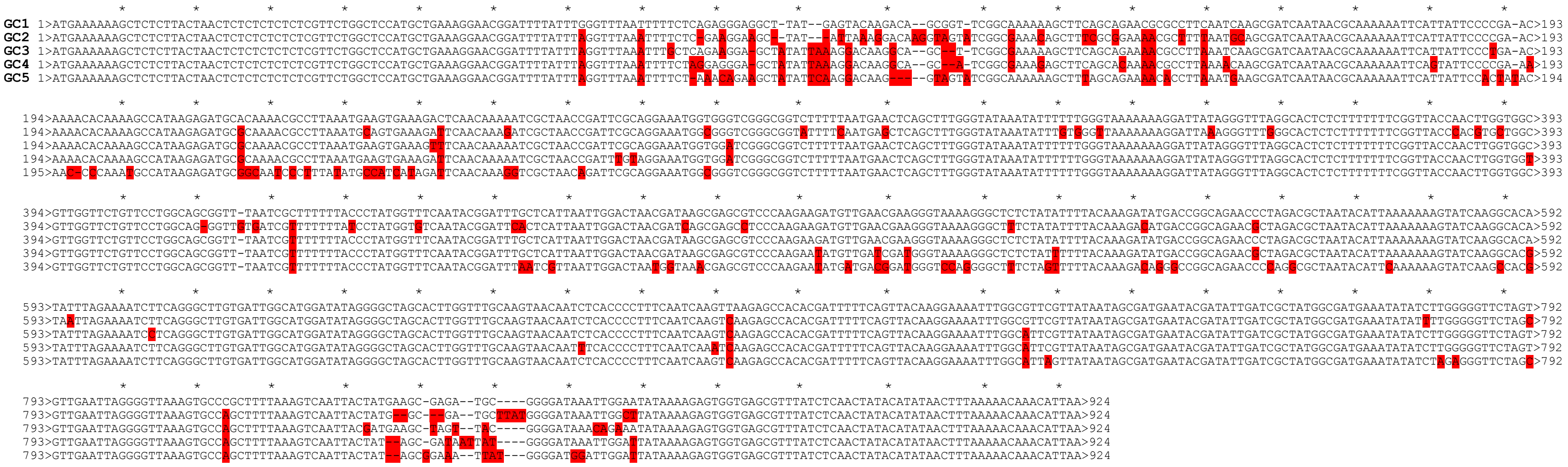

Supplemental Figure 2. Genotyping of the OipA gene in the 5 GC strains. The sequence of OipA gene in the clinical GC strains GC1-GC5 are compared. The nucleotides in red indicated difference and the "-" in sequences denoted a gap.

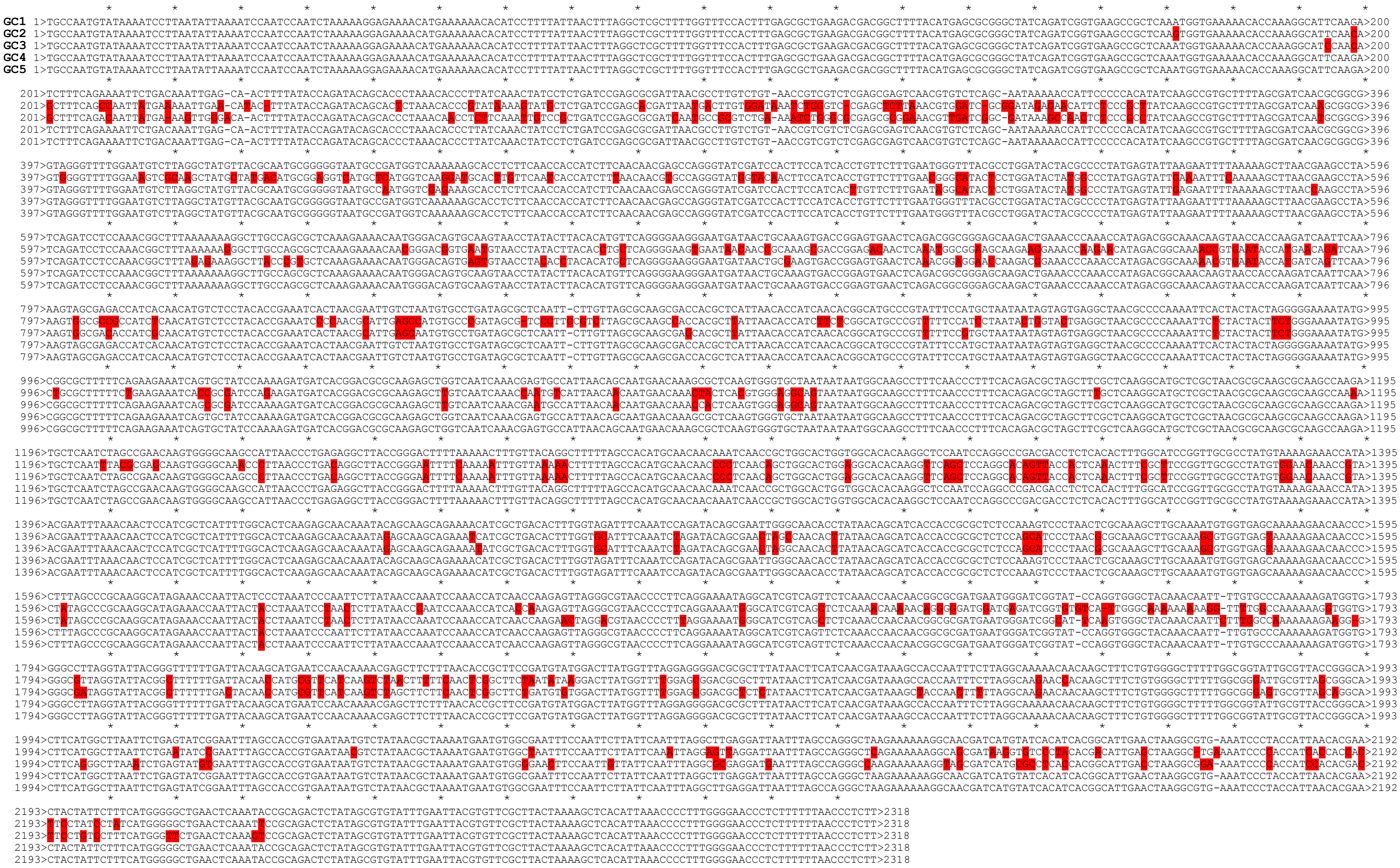

Supplemental Figure 3. Genotyping of the BabA gene in the 5 GC strains. The sequence of BabA gene in the clinical GC strains GC1-GC5 are compared. The nucleotides in red indicated difference and the "-" in sequences denoted a gap.

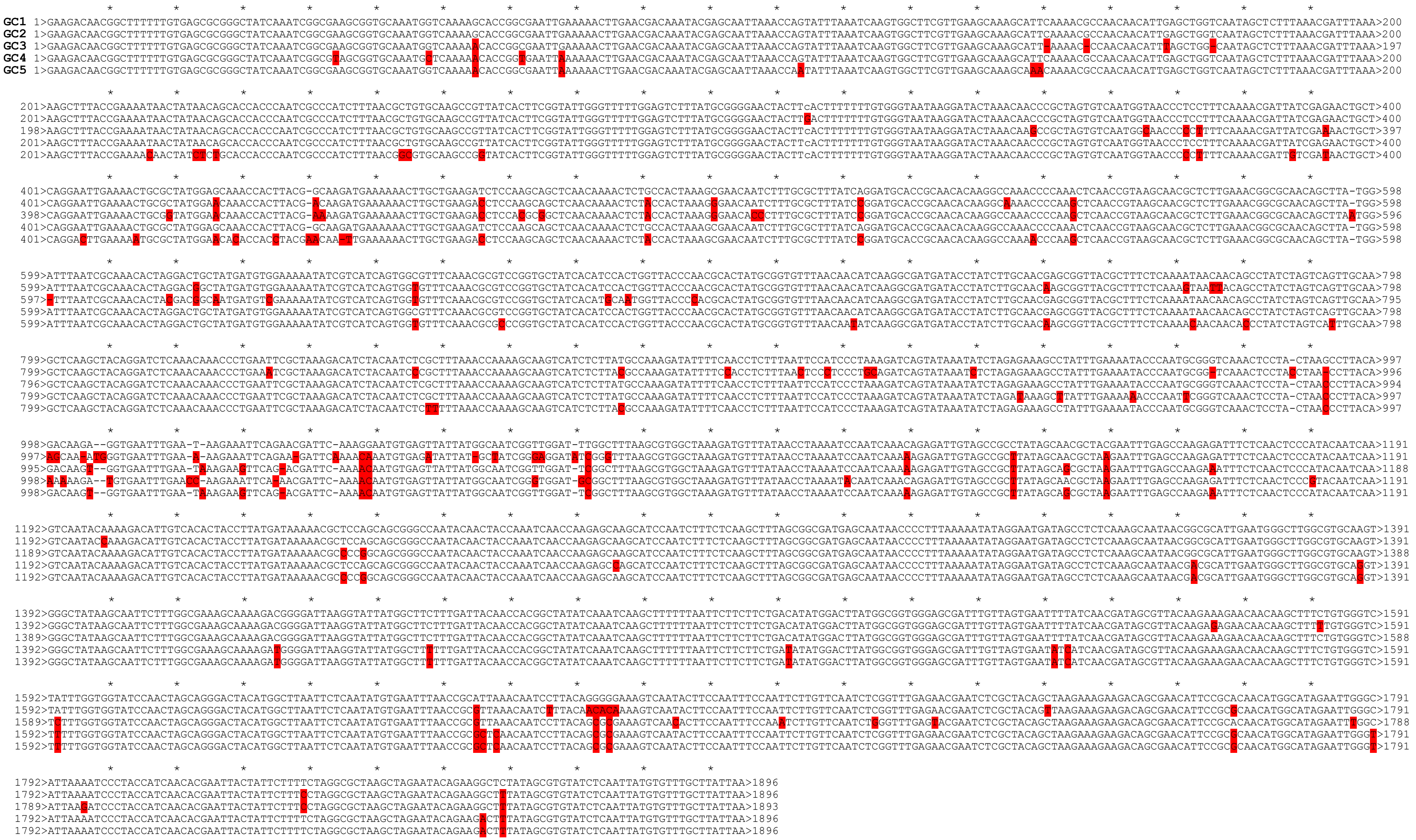

Supplemental Figure 4. Genotyping of the SabA gene in the 5 GC strains. The sequence of SabA gene in the clinical GC strains GC1-GC5 are compared. The nucleotides in red indicated difference and the "-" in sequences denoted a gap.

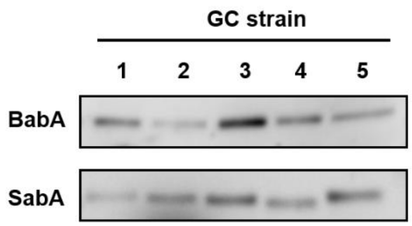

Supplemental Figure 5. BabA and SabA protein expression of the 5 GC strains. Protein extracts from 5 GC-derived *H. pylori* samples were prepared and protein levels of BabA and SabA were examined by Western blotting. The data shown are representative of the results obtained in three independent experiments, all gels and blots were run in the same experimental conditions.

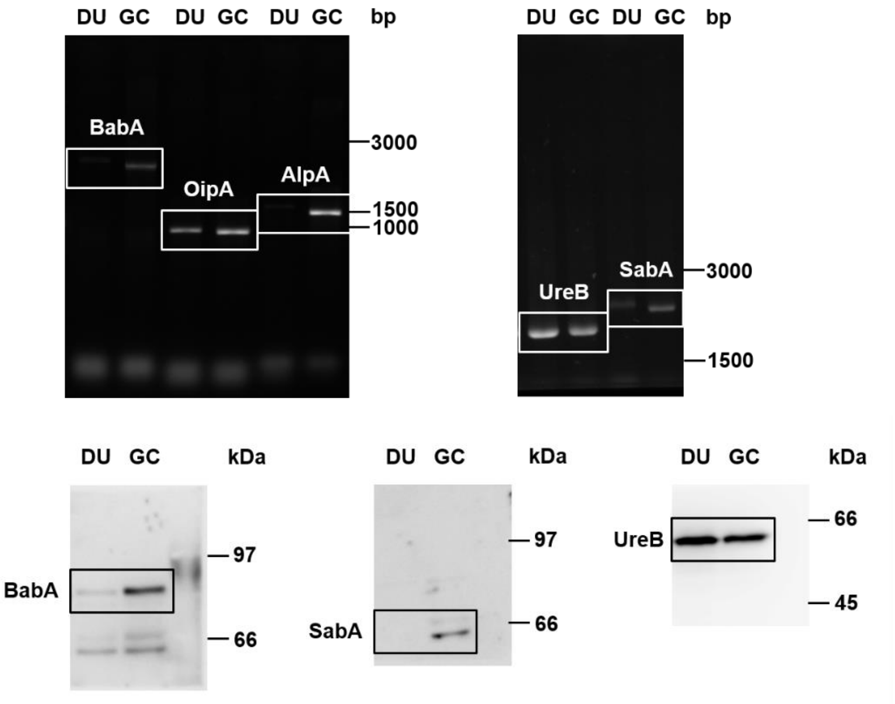

Supplemental Figure 6. Uncropped gels and blots of Figure 2B.
